# Early insights into eyeblink conditioning using optically pumped magnetometer-based MEG

**DOI:** 10.3389/fnhum.2025.1638751

**Published:** 2025-09-24

**Authors:** Chin-Hsuan Sophie Lin, Tim M. Tierney, Stephanie Mellor, George C. O’Neill, Sven Bestmann, Gareth R. Barnes, R. Chris Miall

**Affiliations:** ^1^School of Psychology, University of Birmingham, Birmingham, United Kingdom; ^2^Melbourne School of Psychological Sciences, University of Melbourne, Melbourne, VIC, Australia; ^3^Department of Imaging Neuroscience, UCL Queen Square Institute of Neurology, University College London, London, United Kingdom; ^4^Spinal Cord Injury Center, Balgrist University Hospital, University of Zurich, Zurich, Switzerland; ^5^Translational Neuromodeling Unit, Institute for Biomedical Engineering, University of Zurich & ETH Zurich, Zurich, Switzerland; ^6^Department of Clinical and Movement Neuroscience, UCL Queen Square Institute of Neurology, University College London, London, United Kingdom

**Keywords:** cerebellum, magnetoencephalography, eyeblink classical conditioning, optically pumped magnetometers, Purkinje activities, wearable MEG

## Abstract

**Introduction:**

There is a profound lack of electrophysiological data from the cerebellum in humans, as compared to animals, because it is difficult to record cerebellar activity non-invasively using magnetoencephalography (MEG) or electroencephalography (EEG). Recent developments in wearable MEG sensors hold potential to overcome this limitation, as they allow the placement of sensors closer to the cerebellum.

**Methods:**

We leveraged the development of wearable optically pumped magnetometers to record on-scalp MEG (OP-MEG) during an established cerebellar learning paradigm—eyeblink conditioning. In four healthy human adults, we first validated that OP-MEG can reliably detect cerebellar responses by examining responses to an air puff stimulus.

**Results:**

Significant responses were observed in sensors positioned over the cerebellar region in all four adults in response to the air puff. We then indirectly tested the hypothesis that these responses reflect the population-level spiking activity of Purkinje cells. The air-puff–evoked responses diminished during the acquisition of conditioned responses, corresponding with previously observed changes in Purkinje cell activity in animals. Additionally, in three out of four participants, we observed a cerebellar evoked response just prior to the peak of the conditioned blink, resembling learning-associated shifts in Purkinje cell response latencies.

**Discussion:**

This study demonstrates that OP-MEG is a viable method for recording cerebellar activity in humans. By bridging invasive animal recordings with non-invasive human neuroimaging, these findings provide further evidence of the cerebellum’s role in human learning.

## Introduction

Compared to animal studies, human cerebellar electrophysiology has remained poorly understood due to the longstanding perception that non-invasively recording cerebellar activity using magnetoencephalography (MEG) or electroencephalography (EEG) is particularly challenging (Andersen et al., 2020). One model case that demonstrates this knowledge gap between animal and human cerebellar physiology is eyeblink conditioning. In the classic delayed eyeblink conditioning procedure, a neutral conditioned stimulus (CS), such as a tone or a light, is paired with a blink-eliciting unconditioned stimulus (US) with the CS-US interval being fixed and the two stimuli co-terminating. Over time, the temporal association between the CS and US is acquired and learning is manifested by a conditioned response (CR): blinks which start during the CS and generally peak at the arrival of US.

Evidence from animal lesion (see Christian and Thompson, 2003; for a review), animal electrophysiology (see Jirenhed and Hesslow, 2016; Ten Brinke et al., 2019; for a review), human lesion-symptom mapping (Timmann et al., 2009), and functional MRI studies (Cheng et al., 2008; Dimitrova et al., 2002; Thurling et al., 2015) all robustly support the involvement of cerebellar cortex (mainly lobule VI) and the interposed nucleus in the acquisition and retention of conditioning, indicating shared functional anatomy in animals and humans. However, direct evidence of neurophysiological changes during conditioning in humans remains largely lacking. Identifying electrophysiological markers of conditioning in humans is relevant because eyeblink conditioning is altered in various neurodegenerative (Van Gaalen et al., 2019; Woodruff-Pak, 2001) and neurodevelopmental disorders (Reeb-Sutherland and Fox, 2015), and impaired eyeblink conditioning often starts in the pre-clinical stage of neurodegenerative disorders (spinocerebellar ataxia: van Gaalen et al., 2019; Alzheimer’s disease: Woodruff-Pak, 2001). Therefore, electrophysiological markers of eyeblink conditioning may enhance our chances of early detection and intervention of these diseases. Moreover, given the wealth of knowledge of eye-blink conditioning in animal studies, it provides a powerful testbed for comparative studies, potentially allowing greater confidence to be placed on the identification of signal sources during human MEG or EEG imaging.

Recent studies have challenged the prevailing view that cerebellar activity is inaccessible with M/EEG. Andersen et al. (2020) reviewed evidence of cerebellar activity in M/EEG, while Samuelsson et al. (2020) used realistic simulations to support its detectability. EEG studies using extended arrays have also detected cerebellar responses during conditioning (Todd et al., 2023). Given the complementary sensitivity profiles of MEG and EEG over the cerebellum (Samuelsson et al., 2020) along with the superior spatial resolution in MEG (Baillet, 2017; Hamalainen et al., 1993), there is a clear value for MEG-focused investigations. On-scalp MEG using optically pumped magnetometers (OP-MEG) offers improved signal strength and flexible sensor placement (Boto et al., 2016, 2017), both of which are beneficial for recordings from deep brain areas such as the hippocampus (Feys et al., 2024; Tierney et al., 2021), and by extension, the cerebellum.

Previously, we recorded baseline (i.e., without conditioning) event-related OP-MEG response from sensors placed over the posterior cranium when administering air-puff stimulation to the eyes, a commonly used US (Lin et al., 2019). At sensor level, the temporal profiles of this posterior response were distinct from somatosensory, ocular and muscular responses. Source analysis indicated the response originated from the cerebellum. The fact that this response was time-locked to US delivery suggested it was likely to be the aggregated post-synaptic response to climbing fibre inputs (called the climbing fibre response, CFR) that signal the US (Albus, 1971). To investigate its physiological nature, we introduced a conditioning phase in this study, by pairing air-puffs (US) with auditory tones (CS) in accordance with classical eyeblink conditioning and recorded changes of evoked OP-MEG signals. We concentrated all available sensors (up to 14 at the time of recording) over the posterior cranium to provide more detailed characterisation of the cerebellar response.

As we hypothesised that our previously observed evoked responses represent the population sum of the CFR, our data analysis and expected results were guided by CFR features found in animal electrophysiology and human EEG recordings. First, we expect reduced amplitudes of the US-evoked response during the conditioning phase as compared to baseline. This reduction should be observable regardless of whether a conditioned eyelid closure occurs (Ohmae and Medina, 2015; Todd et al., 2023), although it is expected to be more pronounced in trials with conditioned blinks (Ohmae and Medina, 2015). Second, we expect to see evoked responses during the CS-US interval of the conditioning phase, reflecting the CS-related CFR after learning (Jirenhed and Hesslow, 2016; Ohmae and Medina, 2015; Ten Brinke et al., 2019). This newly acquired CS-related response would be more robust in trials with than without conditioned blinks (Nicholson and Freeman, 2003). Third, we expect to observe pre-CR evoked responses when aligning MEG data to the onsets of CRs on a trial-by-trial basis because the CS-related CFR was found to temporally correlate with CR onset in animal recordings (Ten Brinke et al., 2015, 2017).

## Materials and methods

### Participants

We made use of bespoke 3D printed scanner-casts to fit each individual (see Methods - OP-MEG data recording for their advantages). These are expensive both in terms of preparation time (subsequent to MRI, printing etc) and raw cost which limited our participant pool. Four healthy adult participants (1 female, 3 male), aged 29–52, took part in the experiment. None had any neurological or psychiatric disorders, except for Participant 4, who reported a history of common migraine, which was inactive during the study and not treated with any medication. Participant 1 had previously undergone one session of paired stimulation approximately 7 months prior to these recordings. Participant 2 had taken part in our earlier study (Lin et al., 2019) and was therefore familiar with the unpaired air-puff and sound-tone stimuli used in the protocol, but naïve to the paired CS–US stimulation used to induce eyeblink conditioning. The remaining two participseleants were naïve to all stimulation conditions.

The protocol was approved by the University College London Research Ethics Committee and the University of Birmingham Research Ethics Committee. Written informed consent was obtained from all participants. The experiments took place at the Department of Imaging Neuroscience at University College London, United Kingdom.

### Eyeblink conditioning paradigm

**Figure 1:**
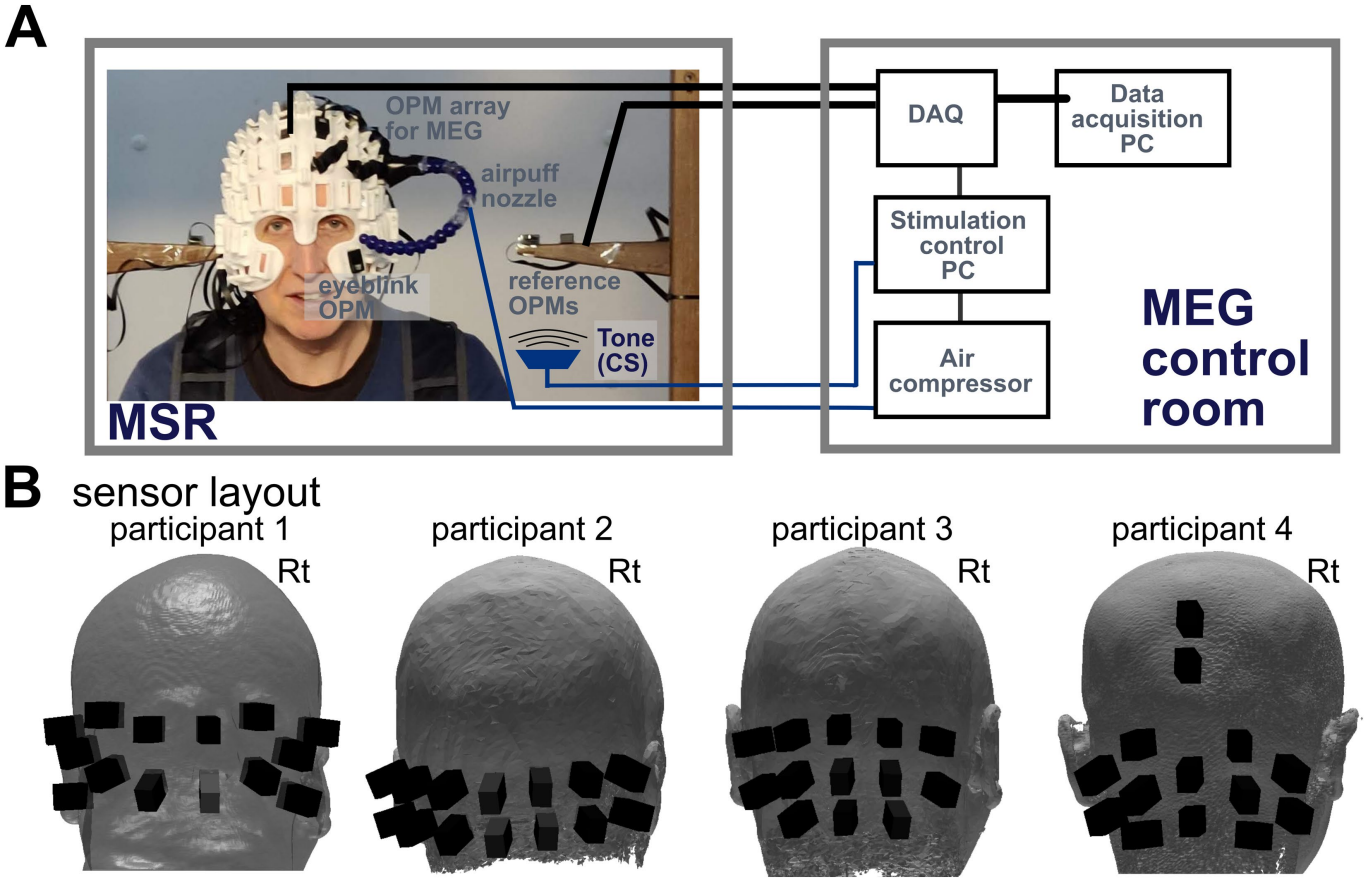
**(A)** Eyeblink conditioning experimental setup. The participant was seated inside a magnetically shielded room (MSR) wearing a customised ‘scanner-cast’. OPM sensors were inserted into slots covering the cerebellum. One additional sensor was placed in the left infra-orbital slot to measure eyeblinks. Air-puffs were delivered to the outer canthus of the participant’s left eye through a nozzle connected to a bespoke air compressor via a rapid action valve. Both the air valve and tone (CS) delivering speaker were controlled by a PC which ran the custom-built experimental code. The data acquisition (DAQ) system recorded and synchronised OPM and trigger signals and then sent data to a data acquisition PC in real time. **(B)** Posterior sensor positions for the OP-MEG recordings. Positions on participant-specific ‘scanner-casts’ were selected that were closest to the cerebellum. Note: Only OPMs used for MEG analysis are shown.

Figure 1A shows the experimental setup. We used a standard delayed conditioning protocol developed by Gormezano and Kehoe, (1975). The US was a brief air-puff delivered through a nozzle mounted on the helmet worn by the participant. The nozzle was connected to a pressurised air cylinder (1 Bar) through a 10 m semi-rigid plastic tube (2 mm internal diameter). Under the control of a bespoke pneumatic valve controller (Leonardelli et al., 2015), the arrival time of the air-puff was estimated to lag the valve opening by 33 ms, calibrated off-line using a microphone, and was relatively insensitive to the nozzle-to-eye distance over a limited range (~1 ms/cm). The nozzle directed the air-puff to the outer canthus of the left eye at approximately 2–4 cm from the eye. The distance was individually set such that the puff was able to evoke a visible blink after each delivery, but without causing discomfort. The CS was a 550-ms tone (2,800 Hz) delivered by a small speaker situated in one access port to the shielded room. There were two phases to each experiment, a baseline and a conditioning phase. Each participant received a baseline block of trials followed by four conditioning blocks. Each block lasted approximately 12 min and the interval between blocks was ~1 min, resulting in a total 1 h of experiment time. The baseline block constituted of 200 trials: 140 US-only trials, 50 CS-only trials, and 10 CS-US paired trials, with the US triggered 500 ms after CS onset. The conditioning block consisted of 190 CS-US trials and 10 CS-only trials (Table 1). In every block, trial order was randomised in sets of 20. Every trial began with a random wait of 1–2.5 s to avoid habituation and anticipation; inter-stimulus intervals averaged to 3.6 s.

**Table 1:** The recording sets consisted of one block of a baseline phase, followed by four blocks of a conditioning phase, with each block comprising 200 individual trials.

Baseline phase 1 block, 200 trials/block	Conditioning phase 4 blocks, 200 trials/block			
140 US-only trials				
10 CS-US trials interspersed	760 CS-US trials interspersed			
	1–190 trials	191–380 trials	381–570 trials	571–760 trials
50 CS only trials interspersed	40 CS only trials interspersed			
	1–10 trials	11–20 trials	21–30 trials	31–40 trials

### OP-MEG data recording

Recording was performed in an OPM-dedicated 4-layer magnetically shielded room (MSR) (Magnetic Shields, Ltd.; internal dimensions 3 m × 4 m × 2.2 m). Participants were seated with their head approximately central within the room during recording. The static field within the central 1 cubic metre of the OPM room is approximately 2 nT and the field gradient is approximately 1 nT·m^−1^ (Mellor et al., 2021). No additional active shielding was used. During the experiment, biomagnetic signals, as well as stimulus timing signals and eyeblink responses were recorded using the OP-MEG data acquisition system. The OP-MEG system for the eyeblink paradigm has been previously described in detail (Lin et al., 2019). Briefly, the system used 15–20 QuSpin zero-field optically pumped magnetometers (QZFM Gen2; QuSpin Inc., Louisville, CO, United States) (Shah et al., 2018; Shah and Wakai, 2013). Each optically pumped magnetometer (OPM) served as one MEG channel, recording electromagnetic signals radial to the scalp. OPMs were mounted on individually 3D-printed rigid sensor helmets (Boto et al., 2017; Meyer et al., 2017; Tierney et al., 2018), with sockets around the outer surface to hold the OPM sensors. The inner surface of each ‘scanner-cast’ helmet was based on an individual participant’s scalp topography extracted from a T1-weighted MRI image. This means sensor positions and orientations were inherently co-registered to individual participant’s brain anatomy. Here, with limited numbers of sensors available, we used the MRI-derived 3D mesh of each participant’s brain surface and of the helmet to select sockets positioned proximal to the cerebellar cortex. Across the 4 participants, 12 to 14 posterior OPM sensors were used. Additionally, one sensor was placed in the left infra-orbital socket to detect eye-blink muscle activity. Due to excessive sensor noise, 2 and 3 posterior sensors were excluded from MEG data acquisition for participants 1 and 4, respectively. This left a total of 12, 14, 13 and 11 posterior sensors for participants 1 to 4, respectively.

Figure 1B shows sensor positions and orientations on the heads. Four additional OPMs (two shown, two not in Figure 1A) were positioned near the head to serve as a reference array, providing measures of the ambient field and interference rejection. OPM signals were recorded using custom data acquisition software built in LabVIEW. Each OPM channel was measured as a voltage (±5 V, 500 Hz antialiasing hardware filter) and sampled at 6 kHz by a National Instruments analogue-to-digital converter (NI-9205, 16-bit, ± 10 V input range) using QuSpin’s adapter.^1^ This signal was then scaled by a calibration factor to represent the recorded magnetic field. The transistor-transistor logic (TTL) signals triggering the air-puff valve and the audio buzzer were acquired simultaneously through the same data acquisition system and sent to the OP-MEG data acquisition PC to align air-puff triggers and OP-MEG data.

### Data analysis

All of the data analysis was performed using SPM12^2^ within the MATLAB environment (Release 2019b, MathWorks Inc., Natick, MA).

*OP-MEG data pre-processing:* OP-MEG data were first filtered between 5 and 80 Hz using a 4^th^ order Butterworth filter. A high-pass filter at 5 Hz was used because artefacts generated by movement of head-mounted OPMs relative to the static gradients in the MSR can be dominant up to 3 Hz. Data were then notch filtered at 50 Hz using a 3^rd^ order Butterworth filter to remove the power line noise. Reference noise cancellation was used to linearly regress the signal recorded by the reference array from the signal recorded at the scalp array (Boto et al., 2017; Fife et al., 1999). For both baseline and conditioning phases, epochs of data for each trial were extracted from −1,000 to +1,000 ms relative to air-puff onset for the US trials, and relative to tone onset for the CS trials. Thereafter, conditioning phase trials were concatenated across the four blocks. Trials from the baseline and conditioning phase were ranked separately according to signal variance, for each participant. These rankings were then used in a median absolute deviation method (Leys et al., 2013) to reject outliers whose variances exceeded three times the median absolute deviation, resulting in an average of 8% trial rejection rate across experimental phases and participants.

*Eyeblink data analysis:* To determine whether and how much each participant blinked, data from the infraorbital OPM were analysed on a trial-by-trial basis using the following custom pipeline. We began with the processed and epoched data, as described in the *OP-MEG data pre-processing* section. The data were then downsampled to 1,200 Hz using SPM12 and low pass filtered at 100 Hz. Thereafter, we performed full wave rectification and low pass filtering at 10 Hz. We then excluded outlier blinks — those with unusually small or large responses — using the same median absolute deviation criterion as for the OP-MEG data, to ensure only reliable responses were included. For both baseline and conditioning phases, the magnitude of the unconditioned eyeblink response (UR) was defined as the peak after US onset. The conditioned eyeblink response (CR) in the conditioning phase was identified using the following amplitude criteria: response onset was automatically detected as being when signal amplitude within the CS–US interval first deviated 3 standard deviations from the pre-CS level in the 500 ms before the CS (Thurling et al., 2015). In trials where pre-CS baseline was not stable and hence no CR was identified by the onset criterion, a CR peak was identified when a response one-twentieth (or greater) of the mean UR magnitude occurred 150–500 ms after CS onset (Woodruff-Pak et al., 1996); responses <150 ms after CS onset were excluded as reflexive responses to the tone (Woodruff-Pak et al., 1996). For a CR that was identified using the peak amplitude criterion, the onset was then identified as the closest trough that preceded the CR peak. All trials were then visually inspected and implausible CRs (such as multiple blinks during the CS-US interval) were excluded. Trials were classified for further analysis as CR negative (CR-) or CR positive (CR+) trials. Trials with no identifiable CR or UR due to unstable pre-CS levels were marked as bad blink trials.

We calculated the percentage incidence and latencies of UR during baseline US-only and conditioning CS-US trials, as well as the percentage incidence of CR during CS-US and CS-only trials across both phases. To assess learning effects, we compared UR incidence and latencies, and CR incidence between baseline and conditioning phases using the Wilcoxon rank sum test. As conditioning progresses, CR incidence is expected to increase from 0 to 1, while UR incidence and latencies are expected to decrease.

*Evoked response analysis:* All the trials surviving blink and OP-MEG outlier rejection were used. For some analyses, CR+ and CR- trials were separated and compared. To identify evoked responses, trials were averaged, and baseline corrected to the mean of the window 100 ms prior to stimulus onset. To identify responses that were potentially time-locked to conditioned blinks (Ten Brinke et al., 2015, 2017), CR+ trial data in the conditioning phase were also re-aligned to blink onset, baseline corrected and averaged. To compensate for the fact that each bespoke cast that houses the OP-MEG sensors has a different layout (Figure 1B), we calculated the global field power (GFP) for participants using the average signal across all sensors for each experimental condition (Lehmann and Skrandies, 1980). Because different participants had very different maxima, either due to individual differences or sensor placement, we normalised each participant’s maximum GFP to 1 to allow for quantitative comparison of global field strengths across conditions. This scaling was applied separately for the baseline versus conditioning comparison and the CR+ versus CR- trial comparison. Given that the number of trials differed across conditions, we employed a 2000-fold unbalanced paired permutation test (Files et al., 2016) to statistically compare normalised GFP amplitudes. Thereafter, we applied a *t_max_* correction (*p* < 0.05) across a 900 ms time window spanning from 117 ms pre-CS to 250 ms post-US to ensure robust control of the family-wise error rate (FWER) (Blair and Karniski, 1993).

*OP-MEG source localisation – beamforming and conjunction analysis*: We used the scalar version of a linear constrained minimum variance beamformer algorithm implemented in the DAiSS toolbox for SPM^3^ to localise the source of evoked responses. The volume conductor model was the Nolte single shell model (Nolte, 2003), implemented in SPM12, using the scalp boundary from the individual T1-weighted MRI. A single covariance matrix was calculated over unaveraged 126 ms post-US window for baseline phase and peri-CR window for conditioning phase, each against a corresponding 126 ms pre-response baseline with a 3–30 Hz bandpass filter. Tikhonov regularization was used with the regularization rate *λ* set to be 5% of largest eigenvalue (Barnes et al., 2004). The source orientation was set in the direction of maximal power. The reconstruction grid spacing was 10 mm. We then performed a conjunction analysis across the MNI-normalised OP-MEG statistical parametric maps. First, we pooled the *p*-values of the statistical maps using the Fisher method described in Heller et al. (2007). We then thresholded for multiple comparisons across the entire brain volume using False Discovery Rate (FDR) with a conservative threshold of *q* < 0.005.

## Results

### Behavioural data

**Figure 2:**
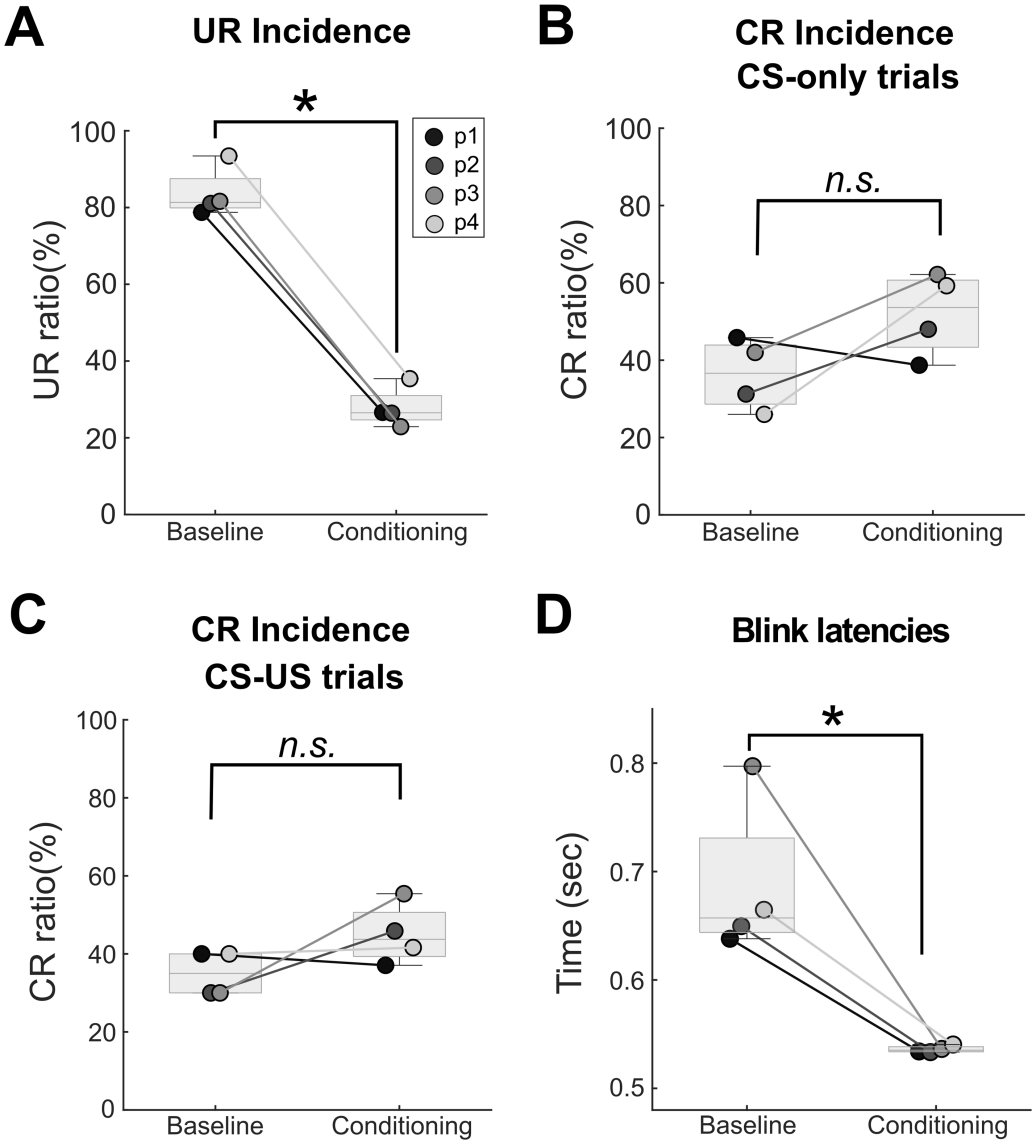
Eyeblink conditioning behavioural data. **(A)** Comparing the UR incidence between baseline US-only trials and conditioning CS-US trials, there was a significant decrease in UR incidence from the baseline to the conditioning phase (median 81 to 27%, one-sided Wilcoxon rank sum test *p* = 0.014). For all the boxplots, the line inside the box is the median of each condition. The lower and upper quartiles are shown as the bottom and top edges of the box, respectively. Individual data points are displayed in participant specific grey dots. “p1” = participant 1 and so on. **(B)** In 3 out of 4 participants, CR incidence during CS-only trials was higher in the last block of the conditioning phase, compared to the baseline. Median CR incidence of CS-only trials increased from 37% during the baseline to 54% in the last block of the conditioning phase although statistically not significant (one-sided Wilcoxon rank sum test *p* = 0.10). **(C)** For the CS-US trials, median CR incidence increased from 35% in the baseline to 44% in the conditioning phase with a marginal significance (one-sided rank sum test, *p* = 0.057). **(D)** Significantly shorter latencies of UR were found in the conditioning phase CS-US trials as compared to the baseline phase US trials. (median latencies: conditioning 0.54 s to baseline 0.66 s, one-sided Wilcoxon rank sum test *p* = 0.014).

Moderate conditioning effects could be observed in the blink responses recorded from the infraorbital sensor. First, in the baseline phase URs were reliably elicited. The median incidence of URs in US-only trials in the baseline phase was 81%, significantly higher than 27% in the CS-US trials in the conditioning phase (Figure 2A; rank sum = 26.00, *p* = 0.014, baseline vs. conditioning 78% vs. 26, 81% vs. 27, 82% vs. 27 and 93% vs. 23% for each participant). Looking next at the CS-only trials, in 3 out of 4 participants, the incidence of CRs was higher in the final conditioning block compared to the CS-only trials within the baseline although statistically not significant (Figure 2B; baseline vs. conditioning 46% vs. 39, 31% vs. 48, 42% vs. 62 and 26% vs. 59% for each participant, median 37% vs. 54%, rank sum = 13.00, *p* = 0.10).

CRs also occurred in about 44% of CS-US trials in the conditioning phase. The median CR incidence during CS-US trials in the conditioning phase was slightly higher than that observed in the limited number of CS-US trials during the baseline period (Figure 2C; baseline vs. conditioning 40% vs. 37, 30% vs. 46, 30% vs. 55% and 40 vs. 42% for each participant, median 35% vs. 44%, ranksum = 12.00, *p* = 0.057). Peak UR latencies were significantly shorter in conditioning CS-US trials than in baseline US-only trials (Figure 2D; baseline vs. conditioning: 0.64 vs., 0.53, 0.65 vs. 0.53, 0.80 vs. 0.54, and 0.67 vs. 0.54 s post-US for each participant, rank sum = 26.00, *p* = 0.014).

In summary, although CRs were higher than expected during the baseline phase and lower during conditioning, there was evidence of associative learning, with increased CRs in 3 out of 4 participants and significant reductions in UR incidence and latencies.

Conditioning phase CS-US trials were further classified based on the presence (CR+) or absence (CR-) of conditioned blinks for evoked response analysis. The number of available trials for each participant was 289, 382, 401, and 296 CR+ trials, and 56, 90, 127, and 97 CR-trials, respectively.

### Evoked responses

**Figure 3:**
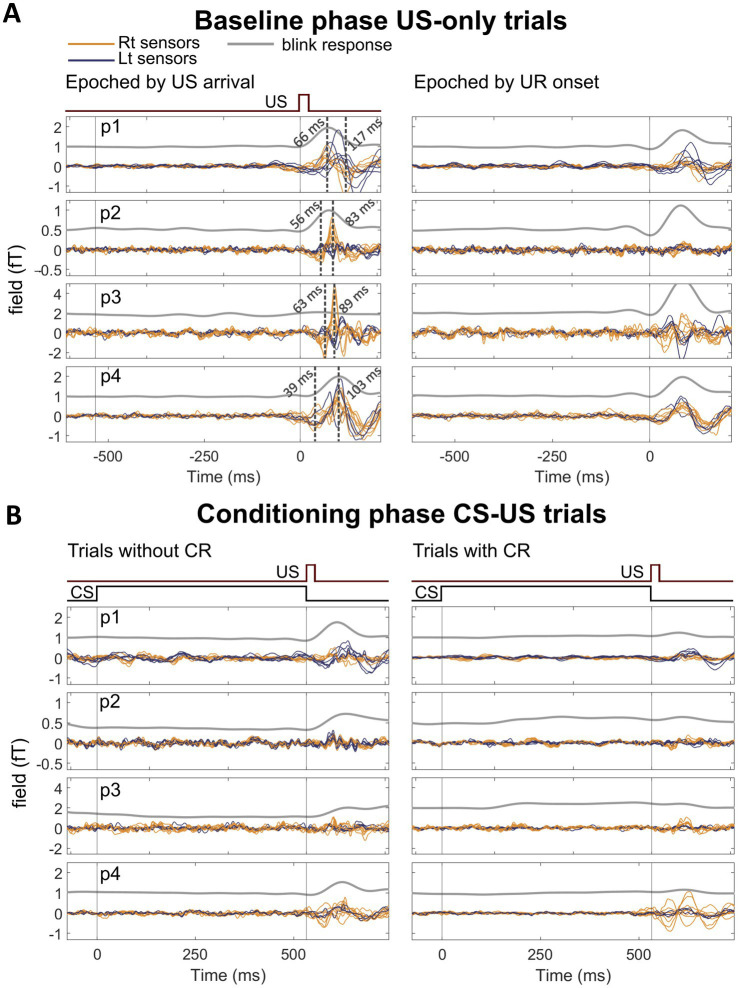
**(A)** Averaged post-US evoked responses (0 ms = US arrival) recorded during the baseline phase. Left panel: trials time-locked to the US onset. Right panel: the same trials time-locked to blink onset. **(B)** Averaged post-US evoked responses recorded during the conditioning phase. Left panel: trials without conditioned blinks. Right panel: trials with conditioned blinks. Each trace corresponds to the average signal from one sensor, including the blink response from the infraorbital sensor (grey line) and the evoked responses from posterior sensors (blue and orange lines represent sensors over the left and right posterior cranium, respectively). Note: the scales of the left y-axis (posterior OP-MEG field strength) differ between participants but are the same between baseline and conditioning phases to best present OP-MEG changes. *p* = participant.

*Baseline phase:* In Figure 3A, left panel, we show the average waveform of US-only trials from OP-MEG data before conditioning. We found two peaks at time windows of 40–65 ms and 80–115 ms after the US in all 4 participants. Their latencies and multiphasic features were largely similar to what we observed previously (Lin et al., 2019). The amplitudes of both early and late peaks did not increase when data were re-aligned to blink onset, suggesting that these responses were stimulus-related rather than blink-related (Figure 3A, right panel). Furthermore, there was no correlation between OP-MEG activity and eyelid position at either early or late time points (Supplementary Figure S1), further supporting the interpretation that post-US evoked responses were driven by the stimulus rather than by blinks.

*Conditioning phase:* Consistent with our prediction, post-US evoked responses were reduced during the conditioning phase compared to baseline (Figure 3B). The reduction can be seen in both the average of CR- trials (Figure 3B, left panel) and CR+ trials (Figure 3B, right panel), suggesting this reduction unlikely to be solely an artifact of the pre-emptive closure of the eyelids. The observed decrease resembles the reduced CFR to the US during conditioning, as reported in animal studies (Ohmae and Medina, 2015). Turning to the CS-US interval, we did not observe a clear peak in our data, except for participant 1 (see Supplementary Figure S3). We speculate that this may be due to differences in the timing of conditioned responses, as further examined in the next section Evoked response preceding CR peaks in conditioning phase.

**Figure 4:**
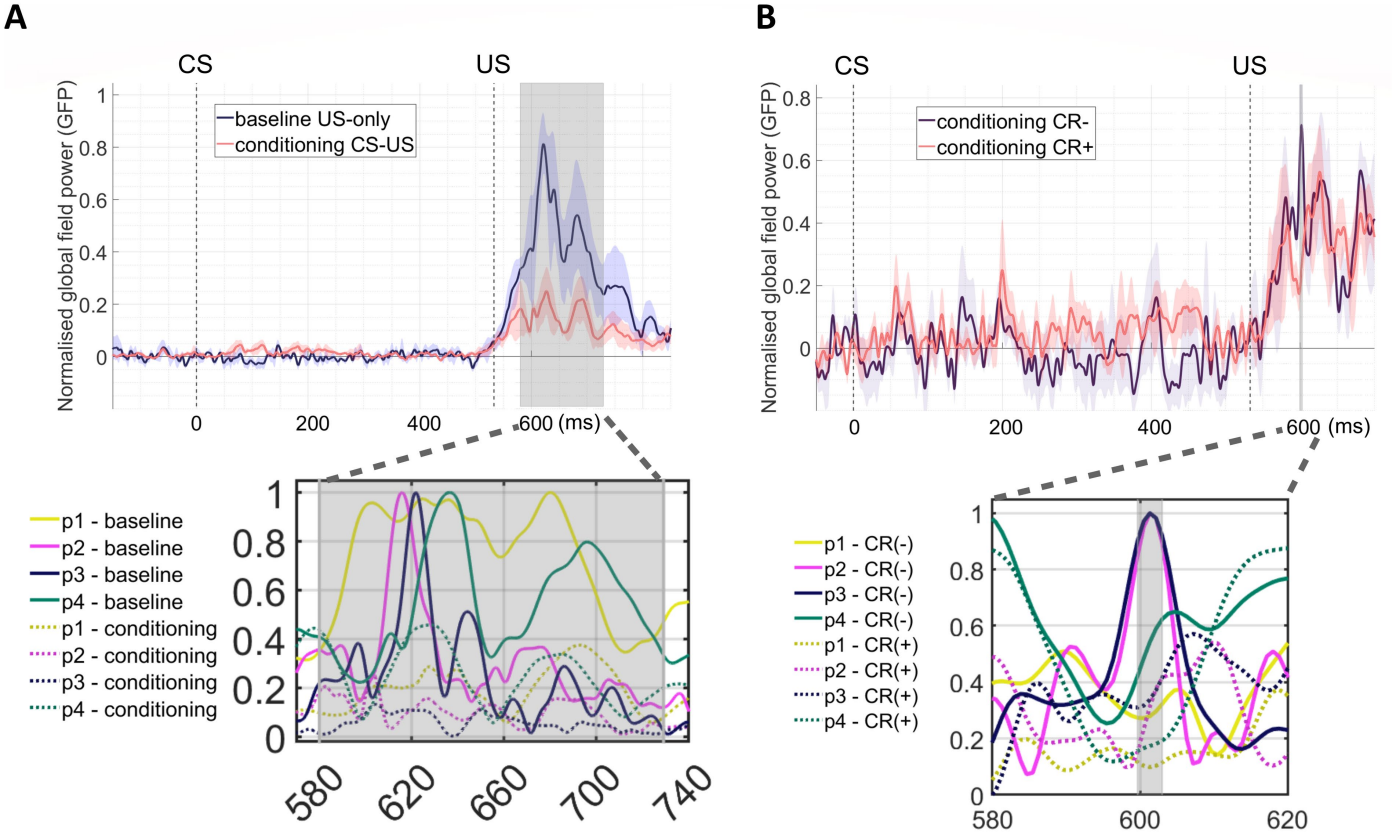
**(A)** Group means of normalised GFPs for baseline (dark blue line) and conditioning (red line) phases with shaded standard error regions. The grey vertical bar indicated significant difference between the two phases as determined by a permutation test, with t_max_ correction for FWER control (*p* < 0.05), showing post-US GFP during the conditioning phase significantly lower than that in the baseline phase between 47 and 196 ms post-US (580–729 ms post-CS onset). The bottom plot zoom into the time window, showing individual normalised GFPs, with solid lines representing baseline data and dot lines representing conditioning data. Note that only US trials were included in the baseline average. **(B)** Group means of normalised GFP for CR- and CR+ trials in the conditioning phase. GFP between 67 and 70 ms post-US (600–603 ms post-CS onset) in CR+ trials (red line) was significantly lower than in CR- trials (violet line), as determined by a permutation test with t_max_ correction for FWER control (*p* < 0.05). The bottom panel zooms into the significant time window, showing individual GFPs, with solid lines representing GFPs of CR- trials and dot lines representing CR+ trials.

We used the grand averaged, normalised GFP to quantitatively assess differences in post-US responses between phases (Figure 4). A 2000-fold permutation test with *t*_max_ correction for FWER control (*p* < 0.05) revealed that post-US GFP was significantly lower in the conditioning phase compared to the baseline phase between 47 and 196 ms post-US (580–729 ms after CS onset, Figure 4A) and also significantly lower in CR+ trials compared to CR- trials between 67 and 70 ms post-US (600–603 ms post-CS onset, Figure 4B). Thus, in the conditioning phase there was a reduced GFP response after the US, contingent on the CRs.

### Evoked response preceding CR peaks in conditioning phase

**Figure 5:**
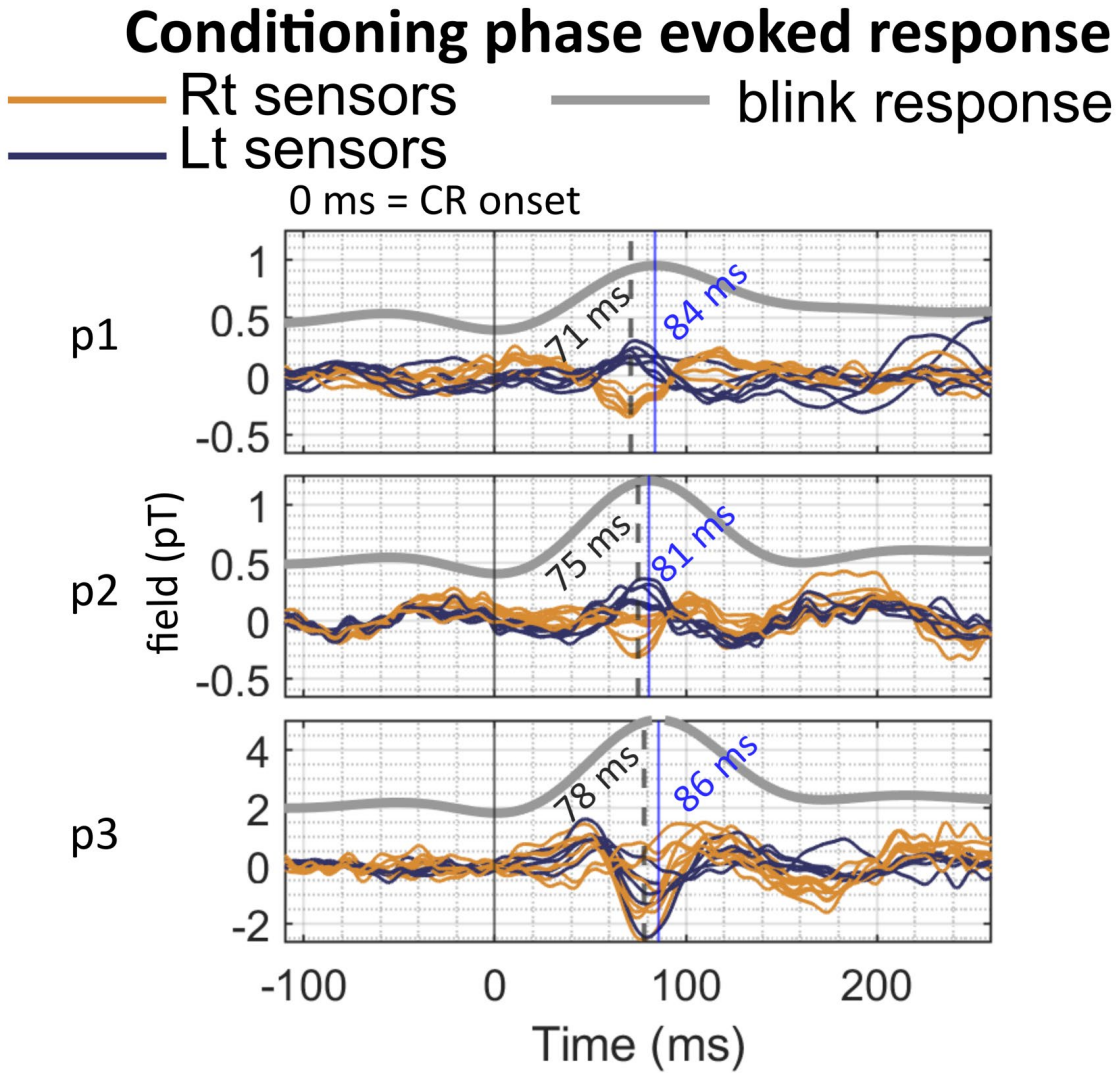
Evoked responses during the conditioning phase. Evoked responses and their respective peak latencies were analysed by aligning trials to the onset of the conditioned response (CR). Each trace represents the average signal from a single sensor located over the posterior scalp, with blue and orange curves indicating sensors positioned to the left and right of the midline, respectively. “p” denotes participant.

Conditioning phase MEG data that were re-aligned to the onset of CRs on a trial-by-trial basis presented with an evoked response peaking at 6–13 ms before the CR peaks in the infraorbital OPM sensor in 3 out of 4 participants, P1-3 (Figure 5). No identifiable peak was found in Participant 4 (Supplementary Figure S5).

### Source level activity of observed magnetic field change

**Figure 6:**
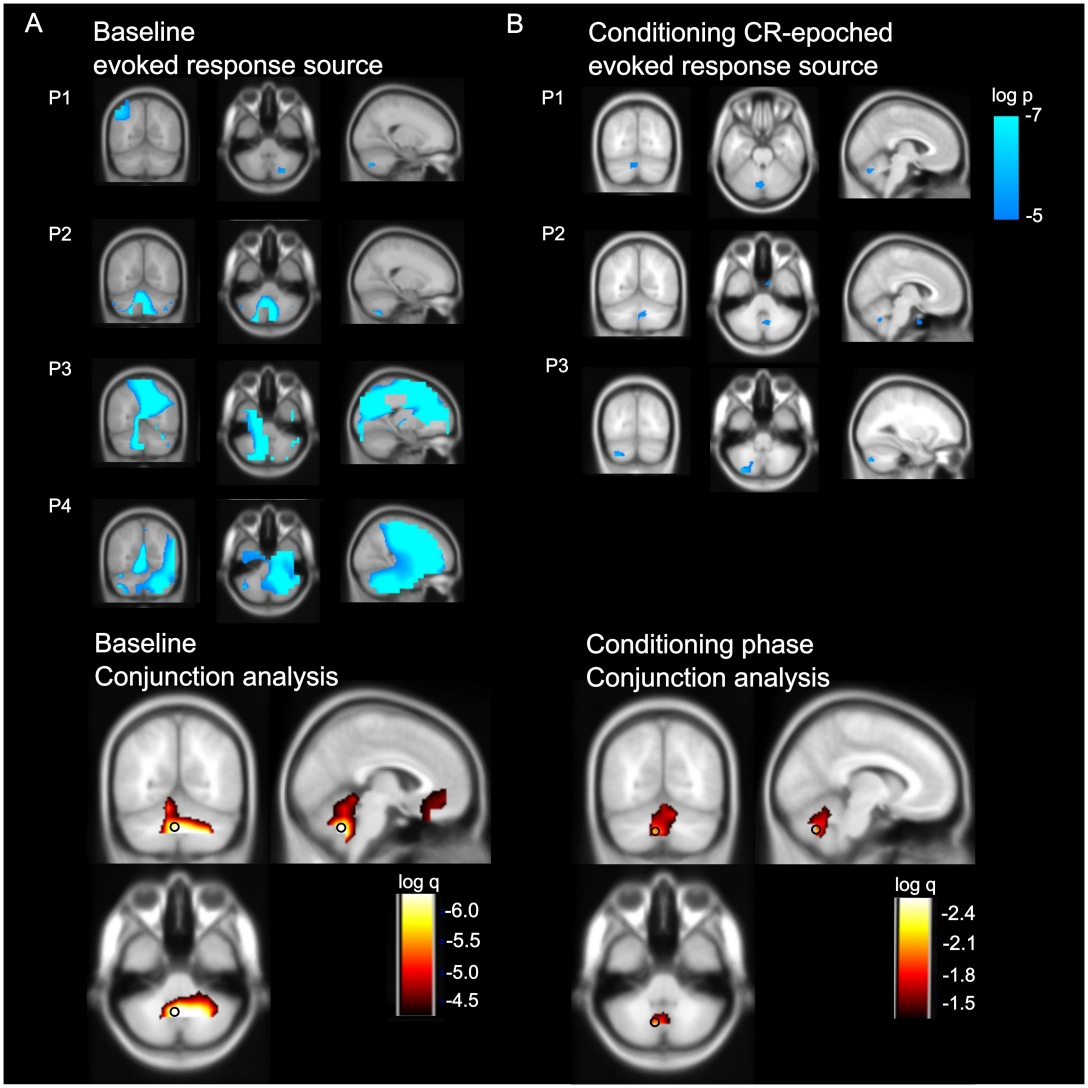
Source level activity **(A)** baseline US-related evoked response **(B)** conditioning CR-onset aligned evoked response Top level: source level data for each participant P = participant. Images are superimposed on the MNI 152 T1 image. Images are thresholded at a significance level of *p* < 0.007 (uncorrected for display purposes). Bottom panel: A conjunction analysis revealed significant activation of the cerebellum for both baseline (left) and conditioning (right) phase activity. The peaks of the cluster were in the left medium cerebellum (indicated with a black circle) for both the baseline (x = −0.4, y = −61.9, z = −43.4) and conditioning (x = −9.3, y = −67.8, z = −41.4) phases. Images of the baseline phase are FDR thresholded q < 0.0001; conditioning phase are FDR thresholded q < 0.05 (only for display purposes).

The top panel of Figure 6 displays changes in evoked power during the 126 ms post-US window (Figure 6A baseline phase) and the 126 ms peri-CR window (Figure 6B conditioning phase), each contrasted with a 126 ms pre-response time window, for each individual participant. A conjunction analysis across participants (Figure 6 bottom panel) revealed a cluster of significant voxels (*q* < 0.005) in the cerebellum with a peak in the left medial cerebellum for both the baseline (x = −0.4, y = −61.9, z = −43.4; vermis VIIIa) and conditioning (x = −9.3, y = −67.8, z = −41.4; left lobule VIIIa) phases.

## Discussion

We recorded OP-MEG from on-scalp sensors placed over the cerebellum during the classic delayed eyeblink conditioning paradigm in 4 participants. Our aim was to extend our previous work (Lin et al., 2019) by examining changes in electrophysiological signals during the acquisition of conditioning, to better infer the neural substrates and functional significance of the evoked response reported earlier. As has been seen previously, we found multiphasic evoked responses to the unconditioned air puff stimulus (US). Beamforming analyses localised the post-US evoked responses likely to the cerebellum. We then showed that this US-related response attenuated during associative learning, i.e., classical conditioning to the CS, and the attenuation was more pronounced in trials with conditioned responses (CR+). In the CS-US interval, during conditioning, we found an evoked response in 3 out of 4 participants when aligning trials to the onset of CRs. Source analysis identified the left cerebellum the most likely origin of this evoked response.

To take these points in turn, the post-US evoked response accords with our previous findings (Lin et al., 2019), with similar latencies of the multiphasic components, and localised within the cerebellum. The peak of the cerebellar cluster was in the vermis VIIIa, a region previously shown to be activated in response to the unconditioned stimulus (US) in an fMRI study of eyeblink conditioning (Thurling et al., 2015). In our previous study, we showed that this response did not originate from the somatosensory cortex or the eyes. As in the previous study, we again found these evoked responses had no temporal correlation with blink responses (Figure 3; Supplementary Figure S1). We believe that the response is most likely the population-sum of the powerful climbing fibre response (CFR) of Purkinje cells, which is characteristic of US-related activity (Ohmae and Medina, 2015). Purkinje cells are the principal neurons in the cerebellar cortex. The large dendritic trees of Purkinje cells align in the sagittal plane of the cerebellar cortex and have a high degree of synchrony within the same functional zone - both features that favour their detectability with MEG (De Zeeuw et al., 2011). MEG recordings from the isolated turtle cerebellum (Okada and Nicholson, 1988) and modelling works on the human cerebellar M/EEG responses (Samuelsson et al., 2020) also suggest that the population activity of Purkinje cells is detectable extracranially. Indeed, we observed diminished post-US evoked response in the conditioning phase and such a reduction was more prominent in trials with conditioned blinks, both in agreement with learning-related reduction in the CFR (Ohmae and Medina, 2015; Todd et al., 2023). Animal data suggests there should also be a conditioned CFR after the CS; we will return to this point shortly.

Questions remain to be answered regarding the nature of some features of US-related responses. One thing that stood out is that we consistently observed multiphasic evoked responses, with early and late peaks evident in each participant (Figures 3, 5A). While the CFRs are multiphasic bursts (Eccles, 1967; Jirenhed et al., 2007; Mostofi et al., 2010; Offenhauser et al., 2005), these bursts are typically only of 5–10 ms duration and unlikely to directly explain the longer 50–100 ms timing of our responses. The other type of US-related activity of Purkinje cells are simple spikes, typically firing at high frequencies, and modulated around a high baseline firing rate. Simple spike firing is also known to be increased by the US (Nicholson and Freeman, 2004) and occurs immediately before the US-driven CFRs (Burroughs et al., 2017). Therefore, it is possible that the earlier component represents the modulation of simple spikes as has been suggested in a previous cerebellar MEG study by Hashimoto et al. (2003), while the later component might reflect the CFR. Indeed, after learning, simple spike suppression during and immediately following the CS-US interval is a hallmark of conditioning (Halverson et al., 2015; Hesslow and Ivarsson, 1994; Kotani et al., 2006; Wetmore et al., 2014), and this direction of change is also consistent with our finding. We, however, acknowledge the challenge of inferring MEG sources by extrapolating from animal single-unit recordings and cannot confirm this with our current results.

Another concern regarding our hypothesis that some components of the post-US response are driven by the CFRs is the latencies of the peaks observed in our studies (this paper and Lin et al., 2019). In small mammal recordings, the latencies of US-triggered CFRs are around 25 ms (Ohmae and Medina, 2015; Ten Brinke et al., 2015) although they could range as far as 60 ms. Some of our responses, especially the late component, occurred much later, at 80–120 ms (Figure 3A, left panel). While the latencies in humans could be considerably longer, due to differences in brain size, our current evidence is not enough to conclude that the US-related evoked response is driven by the CFR per se. Future studies are needed to further understand the neuronal sources of the response.

There were significant between-participant differences in the spatiotemporal distributions of these US-related responses (Supplementary Figure S4). Comparison is challenging because the sensor layouts (Figure 1B) on individual scanner-casts differed substantially; additionally, the number of sensors available was low, hence spatial resolution is low. We calculated the average waveform from a single sensor in each dataset, chosen to have similar on-scalp position across participants and these responses were largely comparable (Supplementary Figure S2). For future studies, we plan to build composite helmets, so that we can maintain comparable sensor layout when recording from different people. By doing so, we can systematically study the spatiotemporal map of the US-related waveform and build norms in the general population.

Turning next to CS-related responses, the CFR has been reported to follow the CS after acquisition of eye-blink conditioning in multiple animal studies (Mostofi et al., 2010; Ohmae and Medina, 2015; ten Brinke et al., 2015), typically with a peak probability somewhat later and broader than that following the US. However, there was no visible peak in the CS-US interval in our data, except in participant 1 (supplementary Figure S3). Several reasons may be behind this result. First, there was only moderate evidence of conditioned behaviour in our experiment, implying learning may have not reached an optimal level. It is suggestive that the increase in post-CS potentials is greatest in participant 1 who had experienced one session of paired CS-US trials several months before. It may be valuable to adopt a pre- and post-conditioning design in future experiments to ensure stronger acquisition of the eye-blink conditioning. Second, learning is marked not only by CS-evoked CFR but also by strong simple spike suppression (Hesslow, 1994; Jirenhed et al., 2007; Rasmussen et al., 2014), followed by a pause at the time of the conditional response and activation of nuclear neurons (ten Brinke et al., 2015). The concurrent early and late responses may weaken signals in extracranial recordings. Third, studies (Rasmussen et al., 2014) have found that few Purkinje cells display enhanced CFR on every trial after learning. Importantly, in their observations, simple spike suppression that temporally proceeded the CS-driven CFRs was strongly correlated with the near absence of CFR in the later time points of CS-US intervals (Rasmussen et al., 2014). Again, our findings raise an interesting question about how the intricate balance between enhanced and suppressed Purkinje cell activity is evident at population level, which is not easily answered by single unit recordings alone.

Next, there were small but significant CR-related evoked responses identified in 3 out of 4 participants, when trial data were aligned to the onset of conditioned blinks. The nature of these remains to be determined. One potential source may be conditioning-related spikes in the nuclear neurons (Rasmussen et al., 2014; ten Brinke et al., 2017). However, the spatial resolution of our source reconstruction is limited so we are not able to differentiate between cortical and nuclear sources in this study. These CR-related responses peaked shortly (< 10 ms) before the peak of the CR eye blink, rather than at CR onset. Taken together with the lack of temporal and amplitude correlations between individuals’ overall OP-MEG peak and their blink data (Supplementary Figure S4), we concluded that the response was not likely to be the CFR. Correlations between neural activity and conditioned behaviours have been identified in background spiking suppression (ten Brinke et al., 2015) and nuclear neuron facilitation (ten Brinke et al., 2017). However, considering all the available evidence, it remains difficult to conclude on the functional implication or the neural generator of these CR-related responses. Last, in our analysis we confined the search for changes below 80 Hz because raw OPM signals beyond 80 Hz displayed high levels of noise. A well-documented change of Purkinje cell activity after conditioning is the suppression of simple spikes during the CS-US interval, maximal just before the CR. A previous EEG study observed reduction of power from beta-band (13–30 Hz), gamma-band (30–80 Hz), and at frequencies up to 320 Hz that may be related to spike suppression (Todd et al., 2019). Although the beta and low-gamma bands fall within the range of our analysis, time–frequency decomposition revealed no significant cerebellar responses in these frequencies (figure not shown). A recent study (Bu et al., 2022) demonstrated that OPMs are capable of detecting frequencies beyond 80 Hz, up to 320 Hz, albeit with weaker signals. Future studies should investigate 80–320 Hz MEG activity for its potential association with simple spike suppression following CR + acquisition.

One might raise the possibility that the cerebellar source locations identified during both the baseline and conditioning phases were artefactual, due to the concentration of sensors over the posterior cranium. We initially used variational Bayesian dipole fitting, which also identified the cerebellum as the best-fitting model for both phases. Although we subsequently used beamforming for prior-free source localisation to improve accuracy (An et al., 2022), we acknowledge that sensor proximity could have biassed localisation toward nearby structures, including the cerebellum. However, in our previous study (Lin et al., 2019), we successfully localised both cerebral and extra-axial sources using beamforming. With that said, we recognise that achieving an optimal trade-off between signal detection and reconstruction accuracy is particularly challenging with a limited number of sensors. Future studies should repeat the measurements using a larger, more evenly distributed sensor array.

To summarise, for the first time we used OP-MEG to identify neural responses that are associated with classic eyeblink conditioning. We found attenuated US-related responses after conditioning, with similarities to the CFR changes observed in animal studies. Although we found no strong evidence for other neural substrates, such as simple spike suppression or nuclear activity, our results demonstrate that OP-MEG is a feasible method for capturing cerebellar activity and provide a critical step toward bridging invasive animal studies with non-invasive human neuroimaging in the study of cerebellar learning.

## Data Availability

The raw data supporting the conclusions of this article will be made available upon reasonable request to the corresponding author.

## Footnotes

^1^ https://quspin.com/products-qzfm/ni-9205-data-acquisition-unit/

^2^ https://www.fil.ion.ucl.ac.uk/spm/

^3^ https://github.com/spm/DAiSS
