# Supplementary material for: Early insights into eyeblink conditioning using optically pumped magnetometer-based MEG
Source: Front Hum Neurosci. 2025 Sep 24;19:1638751. doi: 10.3389/fnhum.2025.1638751 (PMC12504232; doi:10.3389/fnhum.2025.1638751)
Supplement: Supplementary file 1 [file Data_Sheet_1.docx]

**Supporting information for**

**Title: Neural activity in human eyeblink conditioning: an optically pumped magnetometer-based MEG study**

Authors: Chin-Hsuan Sophie Lin, Tim M Tierney, Stephanie Mellor, George C O’Neill, Sven Bestmann, Gareth R Barnes, R Chris Miall

Supplementary figures

**Supplementary Figures**

**
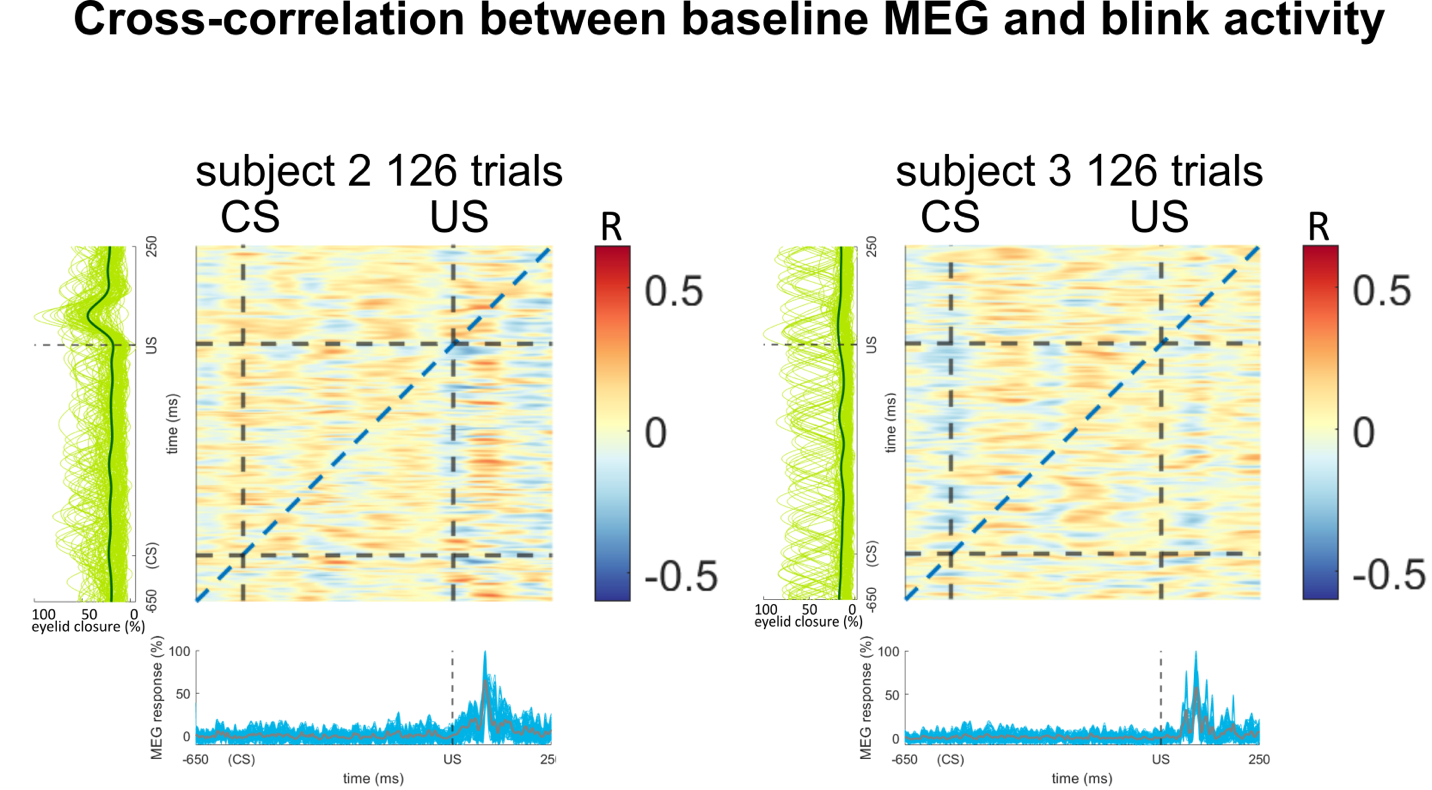
**

**Figure S1** Correlation matrices showed between-trial MEG (x-axis) and blink (y-axis) data interdependence at each timepoint between -650~250 ms, relative to US onset in the baseline phase (see Supplementary Fig S4 for the conditioning phase data). We used rectified data from each subject-specific maximum MEG channel to compute correlation matrices. Single-trial MEG (cyan curve) and average MEG (brown curve) waveform are presented horizontally, below the correlation matrix. Single-trial (light green) and average (dark green) blink trace are presented vertically to the left of the correlation matrix of each participant. Here data from participants 2 & 3 are shown, the correlation matrix of participants 1 & 4 are similar. Strong blink related responses in the MEG data would show up as red areas along the diagonal line, which is not observed in any of our participants. The dashed vertical and horizontal lines indicate the CS-onset (-533 ms) and US-onset (0 ms).


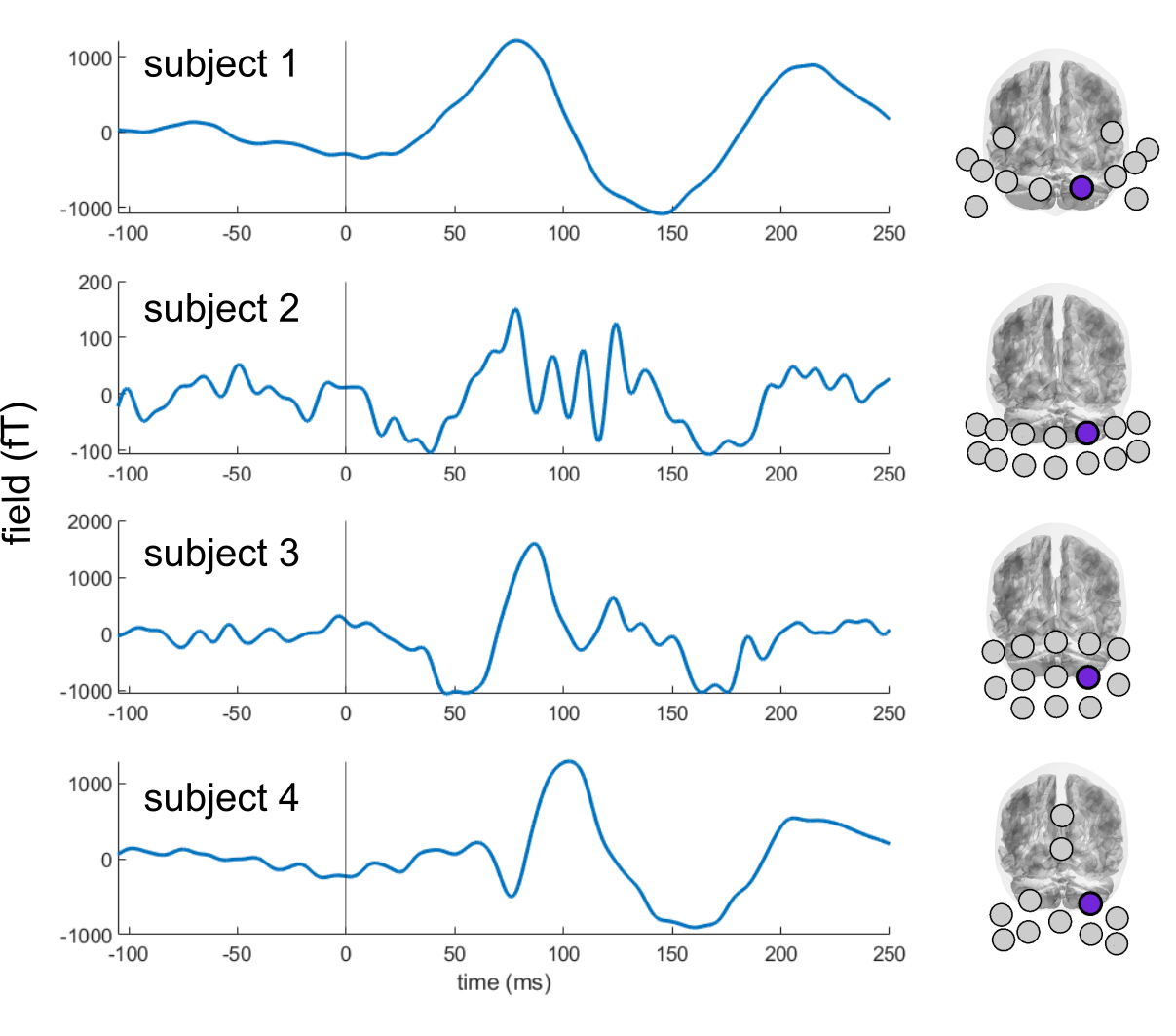


**Figure S2** Average waveforms of baseline (US only) MEG data from one channel of each participant. Channels were selected based on their sensor positions, to be close to the same locus of right cerebellar cortex, as seen in the right panel in blue circles. The time axis is relative to US onset. A positive response peaked between 50-100 ms and a later negative response peaked between 150 – 200 ms were observed in all four participants.

**
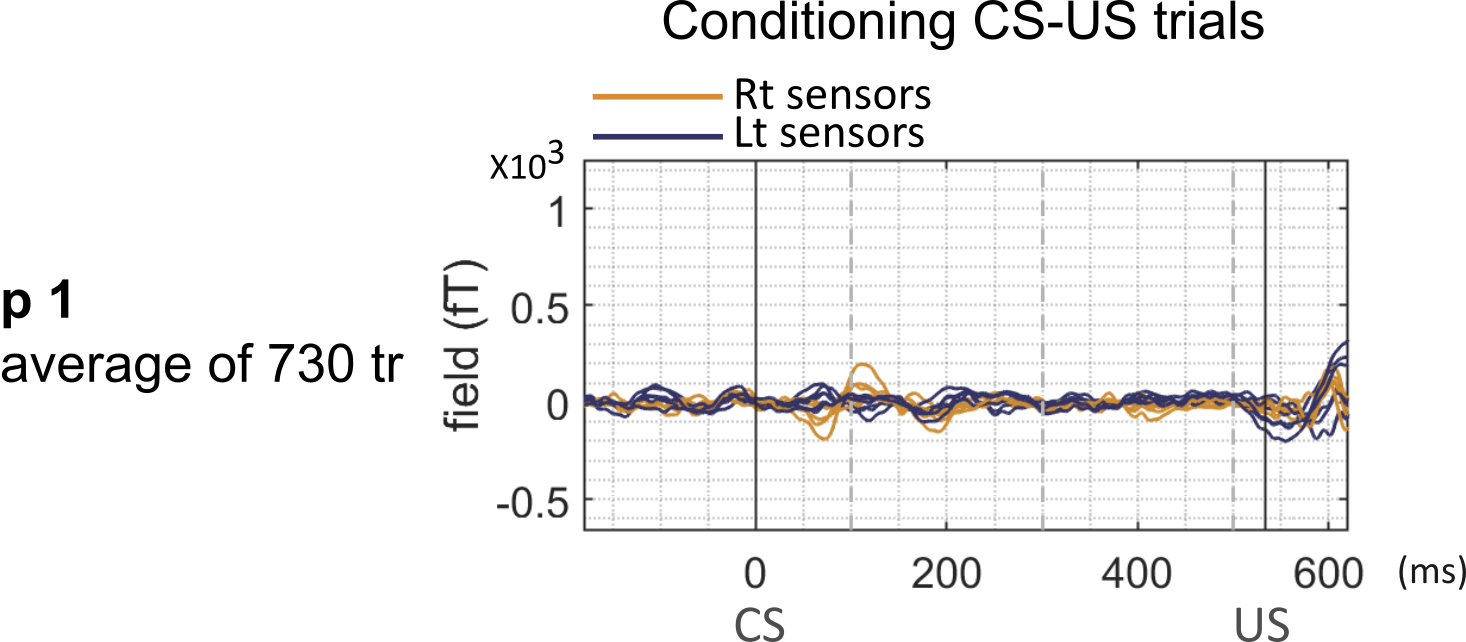
**

**Figure S3** The OP-MEG of all conditioning phase CS-US trials in participant 1 (p1) aggregated and averaged. Two dipolar responses were seen 50-150 ms post-CS. i.e. at a time window similar to baseline evoked US-driven responses. Each trace is an average of of one sensor, across 730 of 760 available trials. To visualize the dipolar activity, the left and right sensors have been colour-coded.

**
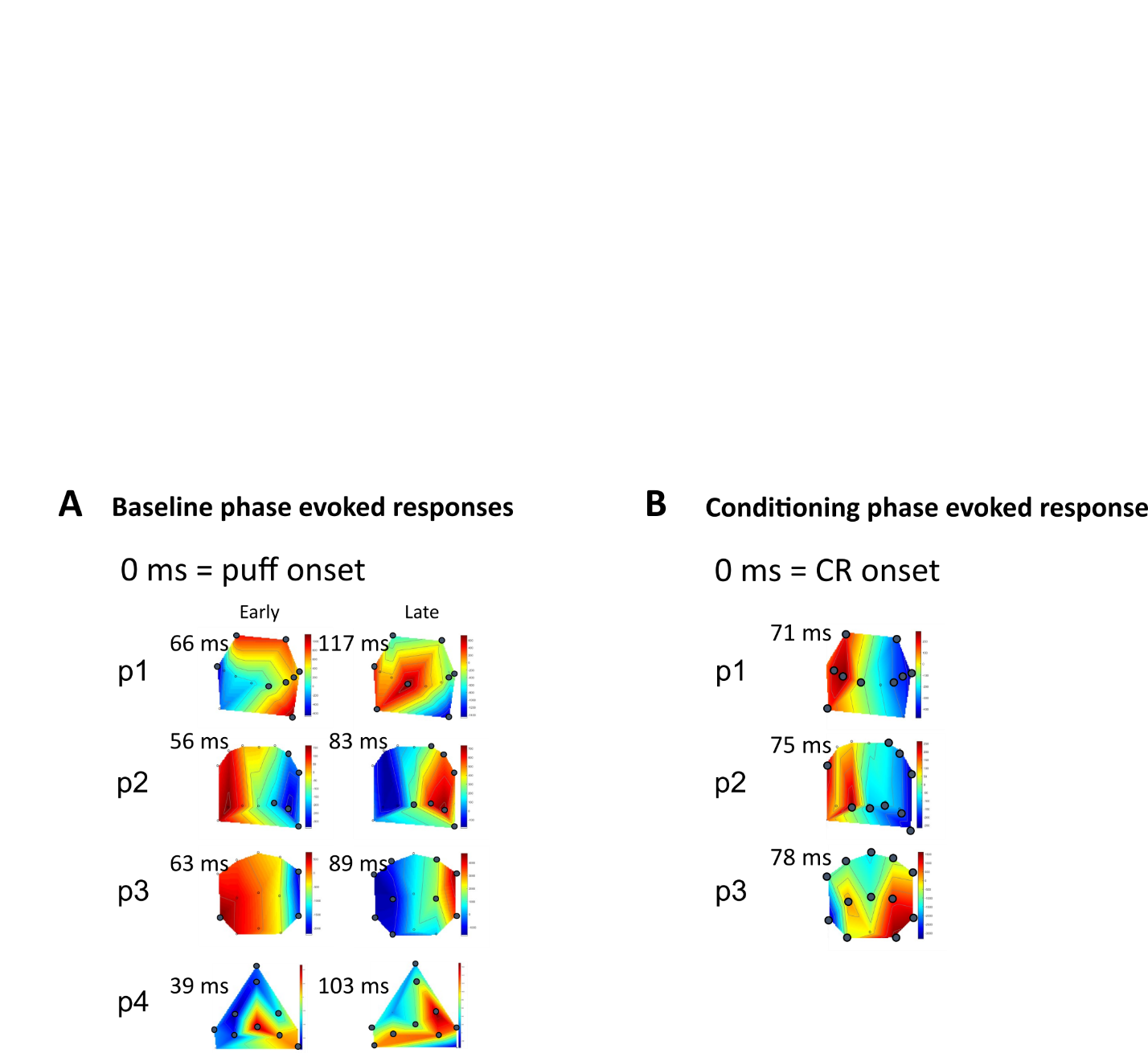
**

**Figure S4 Field maps of evoked responses at individual-specific peak latencies during the baseline and conditioning phases. Sensors showing statistically significant responses compared to baseline are marked with black dots. A. Baseline phase, where MEG data were epoched to the time of puff delivery.** The evoked fields at the participant-specific latencies of the early and late peaks are displayed. For the early peak, significant responses were identified in 8/12, 5/14, 3/13 and 9/11 sensors for each participant. For the late peaks, significant responses were identified in 7/12, 7/14, 9/13 and 9/11 sensors for each participant. **B. Conditioning phase, where MEG data were epoched to the onset of conditioned blinks.** Statistical significance was determined using paired t-tests, comparing mean amplitudes within a ±2 ms window around the peak latency against a baseline window from −4 ms to 0 ms (Bonferroni-corrected, p < 0.05).

**
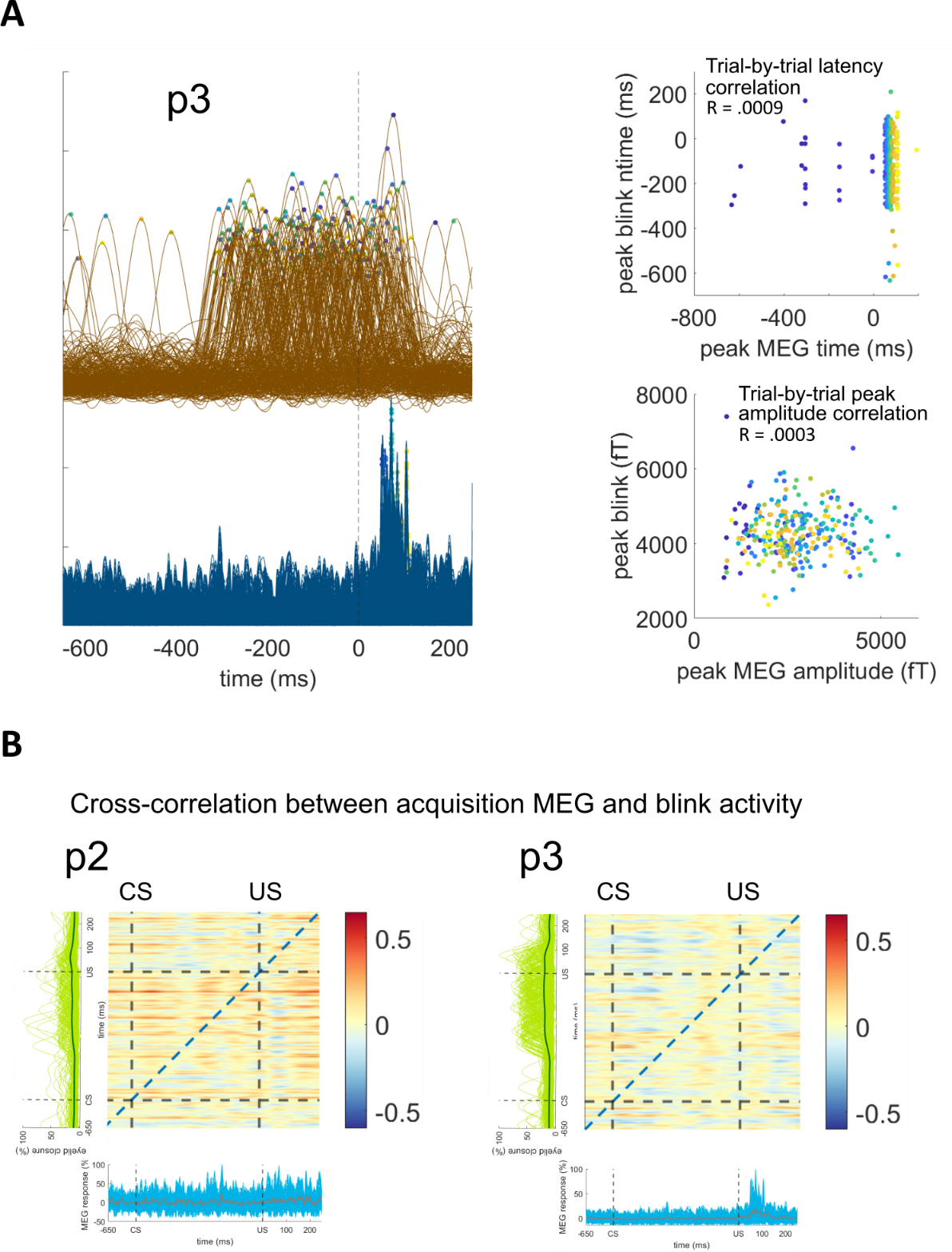
**

**Figure S5 A** CR(+) single trial data of eyeblink (brown curves) and MEG global field power (blue curves) in the conditioning phase, Participant 3. There was no trial-by-trial correlation of peak latency (right upper panel) nor of peak amplitude (right lower panel). The single trial data points in the right panels are colour coded by MEG latency (see upper panel). **B** Correlation matrices show between-trial MEG (x-axis) and blink (y-axis) data interdependence at each timepoint between -650~250 ms relative to US onset, conditioning CR(+) trials. Participants 2 & 3’s data are presented here. Rectified data from each subject-specific maximum MEG channel was used to compute correlation matrices. Strong correlation between MEG and blink responses would present as red areas above the diagonal line, which is not observed in our participants.


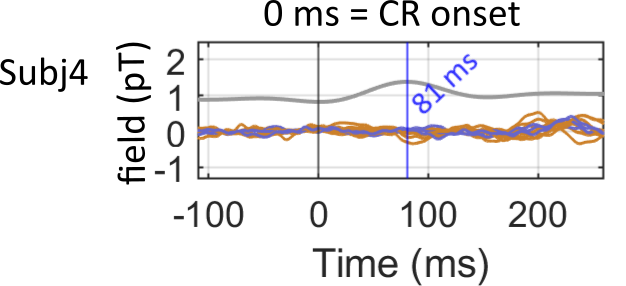


**Figure S6** Participant 4’s average MEG and blink responses of CR+ trials in the acquisition phase. Trial data were aligned to the onset of CRs as for the other three participants (**Figure 5C, main text)**. Each colour trace corresponds to the average signal for one sensor over the posterior cranium, situated left (blue curves) and right (orange curves) of the midline respectively. Average blink peaked at 81 ms (blue text). No MEG peak was found around blink peak latency, different from what was seen in the data of three other participants (**Figure 5C**).
